# Predictors of renal replacement therapy following isolated coronary artery surgery: a retrospective case–controlled study

**DOI:** 10.1097/JS9.0000000000001772

**Published:** 2024-06-26

**Authors:** Alena Krauchuk, Tomasz Hrapkowicz, Piotr Suwalski, Bartłomiej Perek, Marek Jasiński, Tomasz Hirnle, Paweł Nadziakiewicz, Piotr Knapik

**Affiliations:** a Department of Anesthesiology and Intensive Therapy, Silesian Centre for Heart Diseases, Medical University of Silesia; b Department of Cardiac, Vascular and Endovascular Surgery and Transplantology, Silesian Centre for Heart Diseases, Medical University of Silesia, Zabrze; c Department of Cardiac Surgery, Central Clinical Hospital of the Ministry of Interior and Administration, Warsaw and Centre of Postgraduate Medical Education, Warsaw; d Department of Cardiac Surgery and Transplantology, Poznan University of Medical Sciences, Poznań; e Department and Clinic of Cardiac Surgery, Wroclaw Medical University, Wroclaw; f Department of Cardiosurgery, Medical University of Bialystok, Bialystok, Poland

**Keywords:** acute kidney injury, coronary artery surgery, in-hospital mortality, postoperative complications, registry

## Abstract

**Objectives:**

Severe acute kidney injury (AKI) requiring postoperative renal replacement therapy (RRT) is associated with increased morbidity and mortality rates following cardiac surgery. Our study aimed to analyze patients requiring postoperative RRT in a population undergoing isolated coronary artery surgery.

**Methods:**

Following exclusions, we analyzed 124 944 consecutive patients in the Polish National Registry of Cardiac Surgical Procedures (KROK Registry), scheduled for isolated coronary artery surgery between January 2010 and December 2019. Patients who underwent preoperative chronic dialysis were excluded from the study. Data of patients requiring postoperative RRT and patients without postoperative RRT were compared.

**Results:**

In the analyzed population, 1668 patients (1.3%) developed AKI requiring RRT. In-hospital mortality among patients with and without postoperative RRT was 40.1 and 1.6%, respectively (*P*<0.001). Patients requiring postoperative RRT had significantly more preoperative co-morbidities and more frequent postoperative complications. Preoperative chronic renal failure and cardiogenic shock were the two most prominent independent risk factors for postoperative RRT in these patients (OR: 5.0, 95% CI: 3.9–6.4, *P*<0.001 and OR: 3.9, 95% CI: 2.8–5.6, *P*<0.001, respectively).

**Conclusion:**

Severe AKI requiring postoperative RRT dramatically increases in-hospital mortality and is associated with the development of serious postoperative complications. The need for postoperative RRT is clearly associated with the presence of preoperative co-morbidities. Preoperative chronic renal failure and cardiogenic shock were particularly related to the development of this complication.

## Graphical abstract

**Figure:**
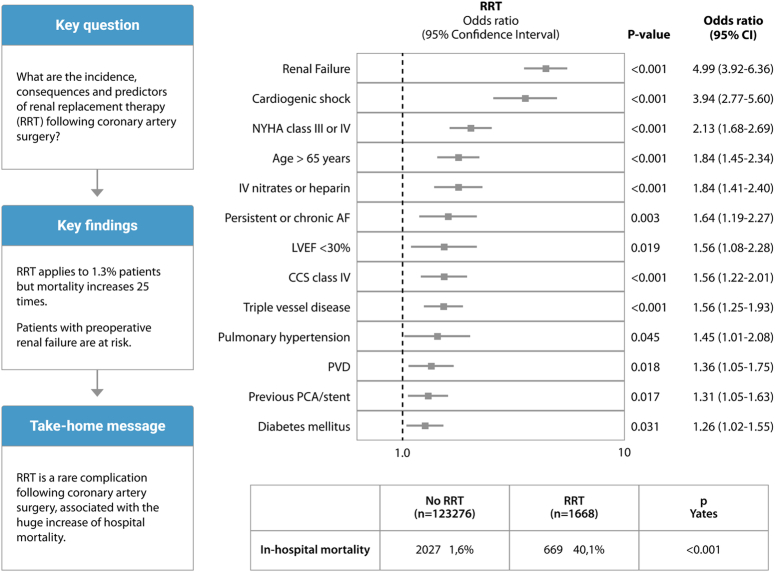

## Introduction

Highlights

- Acute renal failure after coronary bypass grafting applies to 1.3% of patients.
- Need for renal replacement (RRT) therapy increases mortality 25 times.
- Chronic renal failure is a prominent independent risk factor for postoperative RRT.
- Preoperative cardiogenic shock is a prominent risk factor for postoperative RRT.

Renal impairment is a relatively common consequence of cardiac surgical procedures^1^. The spectrum of complications ranges from minor reduction in renal reserve to acute renal failure (ARF). In a short term, acute kidney injury (AKI) is associated with the increased: duration of hospital stay, risk of infection, resource utilization, and mortality^2^. It has been confirmed that even minor postoperative elevations in serum creatinine levels are associated with a significant increase in long-term mortality^2^.

The incidence of ARF after coronary artery bypass graft (CABG) surgery varies from 1 to 5%, depending on the definition of ARF used^3,4^. In patients requiring dialysis or renal replacement therapy (RRT), ARF may be associated with much higher mortality reaching 60%^3^, however, according to the more recent studies this could be reduced to 30–35%^5,6^.

Acute tubular necrosis is the main pathology associated with the ischemia-reperfusion injury involved in the development of AKI in the cardiac surgical population^7^. There are no efficacious treatments to treat established AKI in these patients so far, apart from the implementation of RRT^8,9^. Preoperative risk assessment could be very helpful, because it could indicate patients at risk and allow for an early adjustment of intraoperative and postoperative management.

It has been known for years that RRT increases mortality and the risk of developing serious complications following cardiac surgery^9,10^. In patients receiving CABG, both preoperative renal dysfunction and the deterioration of postoperative kidney function have been proved to be independent predictors of morbidity and mortality^11,12^, however, this issue has only been studied in the homogenous groups of patients following isolated coronary artery bypass surgery (CABG)^1,5^. Nicoara *et al*.^5^ found that in the United States between 1988 and 2003, the incidence of ARF associated with CABG doubled, while at the same time, the associated mortality rate decreased by one-third^5^. Moreover, as mentioned above, we observe conflicting results in terms of the outcomes, as most of the available studies are based on single-center experience^13,14^. In addition, there are also no recent data from Central-Eastern Europe, where a significant number of cardiac surgical procedures is currently performed^15^. Our study, based on the results coming from a large Polish nationwide cardiac surgical registry, was aimed to shed some light on this issue.

### Aim of the study

In the current retrospective analysis, we aimed to compare patients following isolated CABG, requiring, and not requiring RRT in the postoperative period and to identify independent risk factors of this complication. Our analysis was based on the data coming from the Polish National Registry of Cardiac Surgery Procedures (KROK Registry).

## Materials and methods

This analysis is based on the data extracted from the Polish National Registry of Cardiac Surgical Operations (KROK Registry), a joint initiative of the Polish Society of Cardiothoracic Surgeons and the Polish Ministry of Health. The personal data of patients were protected in accordance with the Polish law. The sophisticated personal data protection system provided by the National Center for Healthcare Information Systems was used during data processing. Details regarding the KROK Registry have been previously described^15^.

The primary aim of our study was to compare preoperative and procedure-related variables as well as the postoperative course and in-hospital mortality between patients who required and did not require RRT in the postoperative period. The secondary aim was to identify independent risk factors for the development of this complication.

Our study included all patients who underwent isolated CABG in Poland between 1st of January 2010 and 31st of December 2019 (10 years). Data were collected between 1st of January 2010 and 31st of December 2019 by the KROK Registry and after decision of the Ethics Committee (PCN/0022/KB1/113/20 from 03 July 2020) therefore approved this study as exempt from review and consent of patients has been waived. We have got access to the data for this research on the 10th of October 2020. The work has been reported in line with the strengthening the reporting of cohort, cross-sectional, and case–control studies in Surgery (STROCSS) criteria^16^.

Overall, 125.575 patients underwent isolated coronary artery surgery in Poland in the analyzed period. Excluded were patients with missing or confusing data on key variables (*n*=49). Patients requiring any form of RRT preoperatively, were excluded (*n*=582). Finally, 124 944 patients (99.5% of the total population who underwent isolated coronary surgery in the previously defined timeframe) were analyzed. In this group, a total of 1668 patients (1.3%) who developed AKI grade 3 according to criteria of the Kidney Disease: Improving Global Outcomes (KDIGO) guidelines^17^ and, required RRT in the postoperative period, were identified.

EuroSCORE II and the variable ‘poor mobility’ was introduced to the KROK Registry in January 2012, and therefore, these two variables were assessed only in 97.802 patients (78.3% of the analyzed population – 78.2% patients not requiring RRT and 80.7% patients requiring RRT). Therefore, a variable ‘poor mobility’ was not included later in a multivariable analysis.

The scope of data obtained from the KROK Registry allowed for the assessment of each patient in the following domains: baseline demographic data, circulatory function, individual risk factors, general condition directly before the procedure, and procedure-related variables (Tables 1 and 2). As previously mentioned, patients with missing or confusing data on key variables were excluded. The remaining categorical binary variables with missing data (<5%) were defaulted to the most common value present in the majority of cases.

**Table 1:** Comparison of preoperative variables among patients who required or did not require RRT following coronary artery surgery.

	All	No RRT	RRT	*P*
Variable	(*n*=124 944)	(*n*=123 276)	(*n*=1668)	Yates
Group of variables				
Age ≥65 years	67 630 (54.1%)	66 373 (53.8%)	1257 (75.4%)	<0.001
Female sex	30 715 (24.6%)	30 193 (24.5%)	522 (31.3%)	<0.001
Circulatory function				
CCS class IV	16 363 (13.1%)	15 892 (12.9%)	471 (28.2%)	<0.001
NYHA class III or IV	14 454 (11.6%)	13 966 (11.3%)	488 (29.3%)	<0.001
Recent MI <90 days	35 570 (28.5%)	34 768 (28.2%)	802 (48.1%)	<0.001
Pulmonary hypertension	314 (0.3%)	288 (0.2%)	26 (1.6%)	<0.001
LVEF <30%	4175 (3.3%)	4015 (3.3%)	160 (9.6%)	<0.001
Previous PCI/stent	35 507 (28.4%)	34 969 (28.4%)	538 (32.3%)	0.001
Persistent or chronic AF	6492 (5.2%)	6292 (5.1%)	200 (12.0%)	<0.001
Left main stem lesion	33 204 (26.6%)	32 599 (26.4%)	605 (36.3%)	<0.001
Triple vessel disease	67 225 (53.8%)	66 166 (53.7%)	1059 (63.5%)	<0.001
Individual risk factors				
Cigarette smoking	21 306 (17.1%)	21 027 (17.1%)	279 (16.7%)	0.746
Hypercholesterolemia	79 398 (63.5%)	78 271 (63.5%)	1127 (67.6%)	0.001
Diabetes mellitus	44 141 (35.3%)	43 341 (35.2%)	800 (48.0%)	<0.001
Arterial hypertension	105 955 (84.8%)	104 430 (84.7%)	1525 (91.4%)	<0.001
BMI >35 kg/m^2^	8529 (6.8%)	8385 (6.8%)	144 (8.6%)	0.004
Renal failure	6789 (5.4%)	6335 (5.1%)	454 (27.2%)	<0.001
COPD	8947 (7.2%)	8783 (7.1%)	164 (9.8%)	<0.001
Past TIA, RIND, stroke	4573 (3.7%)	4464 (3.6%)	109 (6.5%)	<0.001
Past treatment of CAD	1413 (1.1%)	1374 (1.1%)	39 (2.3%)	<0.001
PVD	16 035 (12.8%)	15 676 (12.7%)	359 (21.5%)	<0.001
Poor mobility*	5570 (4.5%)	5344 (4.3%)	226 (13.5%)	<0.001
Condition before the procedure				
Cardiogenic shock	3061 (2.4%)	2831 (2.3%)	230 (13.8%)	<0.001
Use of IABP	1434 (1.1%)	1330 (1.1%)	104 (6.2%)	<0.001
IV nitrates or heparin	13 819 (11.1%)	13 344 (10.8%)	475 (28.5%)	<0.001
Procedure-related variables				
Previous cardiac surgery	1894 (1.5%)	1846 (1.5%)	48 (2.9%)	<0.001
Nonelective surgery	46 244 (37.0%)	45 294 (36.7%)	950 (57.0%)	<0.001
CABG	66 275 (53.7%)	65 271 (53.6%)	1004 (61.2%)	<0.001
OPCAB	53 335 (43.2%)	52 722 (43.3%)	613 (37.4%)	<0.001
MIDCAB	3281 (2.7%)	3268 (2.7%)	13 (0.8%)	<0.001
Hybrid (+PCI)	539 (0.4%)	529 (0.4%)	10 (0.6%)	0.378
Conversion to on-pump	620 (0.5%)	569 (0.5%)	51 (3.1%)	<0.001

**Table 2:** The incidence of postoperative complications among patients who required or did not require RRT following coronary artery surgery.

	All	No RRT	RRT	*P*
Postoperative complications	(*n*=124 944)	(*n*=123 276)	(n=1668)	Yates
Neurological complications	1675 (1.3%)	1501 (1.2%)	174 (10.4%)	<0.001
Respiratory complications	3711 (3.0%)	3086 (2.5%)	625 (37.5%)	<0.001
Gastrointestinal complications	785 (0.6%)	613 (0.5%)	172 (10.3%)	<0.001
Renal complications	1442 (1.2%)	811 (0.7%)	631 (37.8%)	<0.001
Sternal, mediastinal or wound infection	2440 (2.0%)	2310 (1.9%)	130 (7.8%)	<0.001
Perioperative myocardial infarction	1425 (1.1%)	1281 (1.0%)	144 (8.6%)	<0.001
Mechanical circulatory support	2577 (2.1%)	2138 (1.7%)	439 (26.3%)	<0.001
Reoperation due to bleeding	4502 (3.6%)	4183 (3.4%)	319 (19.1%)	<0.001
In-hospital mortality	2696 (2.2%)	2027 (1.6%)	669 (40.1%)	<0.001

Patients who developed postoperative complications in the analyzed cohort were identified. Neurological, respiratory, renal, gastrointestinal, and infectious complications were defined according to the rules previously described in the other studies coming from the KROK Registry^15^.

### Statistical analysis

Initially, patients requiring RRT in the postoperative period were compared to the remaining population. Continuous variables were presented as mean and SD, while categorical variables were presented as percentages. *χ* ^2^ test, Mann–Whitney *U* test, and Student’s *t*-test were used to test for statistical significance, where appropriate.

The independent demographic, preoperative, and procedure-related variables (listed in table I) were compared between patients who required and did not require RRT in the postoperative period. The effect of independent variables on ICU readmission was calculated by means of univariable logistic regression. Variables with a *P*-value <0.05 were then included in the multivariable logistic regression analysis, where *P*<0.05 was considered as significant.

For all analyses, a two-tailed *P*-value <0.05 was considered statistically significant. The analyses and graphs were performed with the use of Dell Inc. (2016). Dell Statistica (data analysis software system), version 13.

## Results

Following exclusions, we analyzed 124 944 consecutive patients in the Polish National Registry of Cardiac Surgical Procedures (KROK Registry), scheduled for isolated coronary artery surgery between January 2010 and December 2019. In the analyzed population, 1668 patients (1.3%) developed AKI grade 3 according to KDIGO with the need for RRT. In-hospital mortality among patients with and without postoperative RRT was 40.1 vs 1.6%, respectively (*P*<0.001).

The mean age of patients with and without the need for postoperative RRT was 70.2+-8.4 vs 65.6+-11.7 years, respectively (*P*<0.001). The female sex was present more often in a group requiring RRT in comparison to the remaining patients (31.2 vs 24.5%, *P*<0.001).

Patients with the need for postoperative RRT had significantly more preoperative co-morbidities. The most significant was more frequent presence of poor circulatory function, including cardiogenic shock and unstable coronary artery disease with main stem lesion. Additionally, patients requiring RRT were more frequent female, diabetic, with peripheral vascular disease, undergoing nonelective surgery. The entire comparison of preoperative variables of patients who required or did not require RRT following coronary artery surgery is presented in Table 1.

Postoperative RRT was linked to more frequent postoperative complications. For example, major neurological complications were increased nine times, respiratory complications – 15 times and severe gastrointestinal complications – more than 20 times. The frequency of perioperative myocardial infarction in these patients was increased ninefold, reoperation due to bleeding – sixfold, and wound infection – fourfold. A comparison of the frequency of postoperative complications among patients who required or did not require RRT following isolated CABG is presented in Table 2.

**Figure 1:**
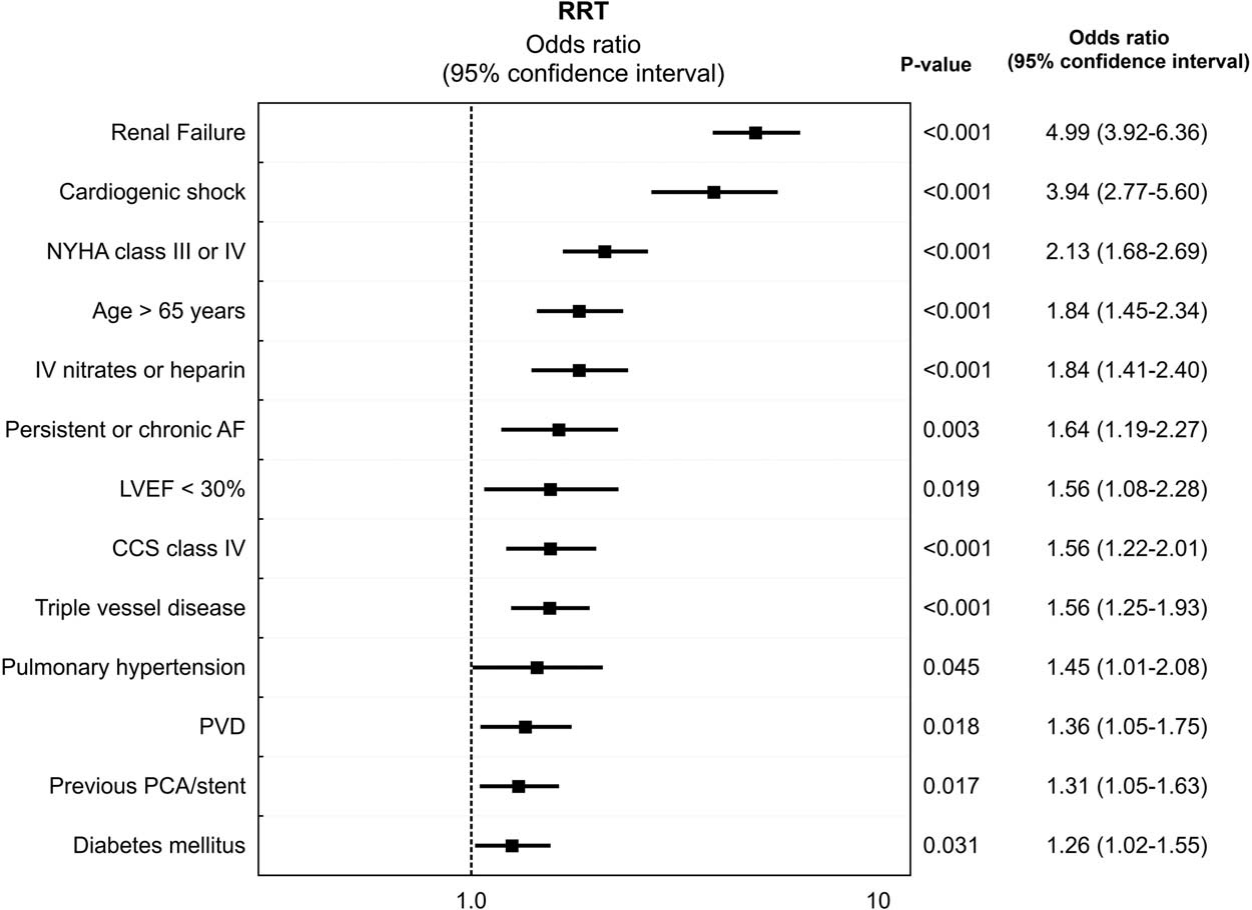
Independent predictors of the need for postoperative renal replacement therapy following coronary artery surgery.

Multivariable analysis was able to identify 13 independent preoperative risk factors for the development of postoperative RRT. Preoperative chronic renal failure and preoperative cardiogenic shock were among the most prominent independent risk factors for postoperative RRT in these patients (OR: 4.99, 95% CI: 3.92–6.36, *P*<0.001 and OR: 3.94, 95% CI: 2.77–5.60, *P*<0.001, respectively). The full results of the multivariable analysis are presented graphically in Figure 1.

## Discussion

In our study, we were able to confirm very high mortality among patients requiring RRT following isolated CABG. Patients with the need for postoperative RRT had significantly more preoperative co-morbidities and more frequent postoperative complications. Preoperative chronic renal failure and cardiogenic shock were the two most prominent independent risk factors for postoperative RRT. Overall, 13 independent risk factors were identified.

Our observations cover the period of 10 years. During this period, there were over 10 000 coronary surgery procedures performed in Poland each year, with a rapid increase of operations performed in the most advanced age group^18^. Studies conducted on such large populations (>100 000 patients) allow for drawing reliable conclusions. The frequently cited landmark study with a comparable sample size (43 642 patients), but more heterogenous population was published by Chertow *et al*.^3^ in the Circulation journal over 25 years ago. More recently, Swaminatham *et al*.^1^ analyzed a large nationwide database covering 20% of US inpatient discharges following CABG between 1988 and 2003 and found a significant decline of in-hospital mortality (from 47.4 to 29.7%) among patients requiring postoperative dialysis^1^. Most recent reports (based on single-center studies) tend to confirm these findings^4,14^. In comparison with these data, in-hospital mortality of our patients requiring postoperative dialysis following CABG was relatively high (40.1%).

It must be remembered, that apart from the largely increased perioperative mortality, postoperative RRT and any impairment of postoperative renal function also affects long-term follow-up results. It has been recently proved, that in the mixed cardiac surgical population, survival among patients with postoperative RRT is significantly reduced at 30-days, 1-year, and 5-years^4^. Mehta *et al*.^19^. discovered that long-term mortality was significantly higher in those patients with ARF who presented with incomplete recovery of renal function^19^. Zakeri *et al*.^20^ showed that patients with mild renal dysfunction have higher perioperative mortality, morbidity and their 3-year survival is adversely affected^20^. In a meta-analysis performed by Corredor *et al*.^21^, it may be clearly seen that a composite hazard ratio for postoperative mortality is significantly elevated in patients with postoperative ARF^21^.

The fact, that the need for postoperative RRT is associated with the development of serious postoperative complications comes as no surprise and the cause-and-effect relationship in this case seems to be obvious. In most cases, the need for postoperative RRT affects patients with significant comorbidities, suboptimal perioperative care, who previously develop other serious postoperative complications. For example, it has been proved that re-exploration for bleeding increases the risk of postoperative RRT more than fivefold^15^. Mathis *et al*.^22^ stated that the intraoperative hypotension is associated with increased risk of AKI in noncardiac surgery.

Patient-related factors are often more strongly associated with postoperative mortality than surgical factors. From a clinical perspective, many of these factors are not modifiable. Preoperative chronic renal failure was the most prominent independent risk factor for postoperative RRT in our cohort. Similar findings may be also found in the medical literature. It has been previously confirmed that AKI requiring preoperative dialysis increase surgical risk more than 20 times^10,23^. In the large STS database analysis, Cooper and colleagues stated that a reduced glomerular filtration rate (GFR) among nondialysis patients was a strong predictor of postoperative RRT^23^. In this analysis, nearly 500 000 patients were divided according to their preoperative GFR. The risk of postoperative RRT was confirmed to be greater among patients with severe, moderate, and even mild (GFR 60–90 ml/min.) renal dysfunction^23^. Some studies have attempted to refine prediction of the occurrence of kidney injury and the need for dialysis, particularly in those patients with pre-existing renal dysfunction^24^.

Among 124 944 patients analyzed in our study, 1.3% developed AKI with the need for RRT in the postoperative period. In a large meta-analysis of all available studies concentrating on this subject, published by Hu *et al*.^24,25^ in 2016, pooled rate of RRT following cardiac surgery was found to be 2.3%. Our overall prevalence of ARF following cardiac surgery is comparable with prior studies^3,4,26^.

The influence of off-pump technique in this context in inconclusive. Another meta-analysis of 22 randomized controlled trials (including 4819 patients) suggested that OPCAB may be associated with a lower incidence of AKI^27^, while the German OPCAB in Elderly Patients Trial detected no difference in the incidence or severity of AKI undergoing OPCAB versus CABG^28^. The advantage of OPCAB technique among patients with severely impaired preoperative renal function was also confirmed in a single center study published by Fuster *et al*.^29^. In our study, we noticed that patients operated off-pump required RRT less frequently in comparison to their on-pump counterparts, however, this factor was found to be significant only in the univariate analysis and did not enter a multivariable model. Li *et al*.^11^ stated that the incidence of AKI with on-pump and off-pump coronary artery bypass was 10.4 and 3.5%, respectively. Apart from that, each 10 min increase in the duration of cardiopulmonary bypass time was found to be associated with an odds ratio of 1.06 for ARF^26^.

Multivariable analysis performed in a group of our patients revealed, that patient’s preoperative hemodynamics and urgency of the surgical procedure can also increase the risk of postoperative RRT. This risk has been already confirmed for the conditions such as CCS class IV angina and acute coronary syndrome with cardiogenic shock that require intra-aortic balloon pump (IABP). The three already available scoring systems (Cleveland Clinic model, Mehta score and the Simplified Renal Index) include these risk factors in their predictive models for postoperative AKI and the need for postoperative RRT^10,30^. The other factors found in our multivariate analysis are also present in these three above mentioned predictive models.

It must be mentioned that there are more predictive models for postoperative RRT in the medical literature. O’Neal *et al*.^31^ proved that more complex procedures, age, sex, and conditions such as arterial hypertension, hyperlipidemia, diabetes mellitus are preoperative risk factors for postoperative RRT. Birnie *et al*.^32^ created the Leicester Score to predict cardiac surgery-associated AKI (CSA-AKI), which showed better discrimination ability when compared to EuroSCORE II and the Cleveland Clinic Score^10^, however, this model included also some other factors such as age, peripheral vascular disease, preoperative GFR, presence of triple vessel disease, ejection fraction, emergency surgery, and type of surgery^32^.

In our study, preoperative renal failure was identified as the most prominent risk factor for postoperative RRT. In view of such an obvious fact, one may ask why this variable has not turned to be the most important risk factor in the other predictive models for postoperative RRT?

This might be due to our definition of this comorbidity. In our study, we excluded all preoperatively dialysis-dependent patients, and the term ‘preoperative renal failure’ was assigned to patients if creatinine clearance was lower than 50 ml/min, (which corresponds to the definition of ‘severe renal impairment’ according to description of the EuroSCORE II predictive model). Therefore, the other group of patients (without this condition) included both patients with normal renal function (creatinine clearance >85 ml/min) and patients with moderately impaired renal function (creatinine clearance 50–85 ml/min). This may explain why preoperative renal failure emerged as the most important risk factor for postoperative RRT in our study (OR: 4.99, *P*<0.001).

Our study was observational, retrospective, and therefore has several limitations. First, we did not stratify patients preoperatively to various degrees of renal failure. Patients with the most severe form of preoperative ARF (preoperative dialysis) were excluded, but the 5.4% of the remaining patients still had various degrees of preoperative renal failure and were included in the study (Table 1). Second, we were strictly limited to the data available in the KROK Registry. For that reason, we were not able to establish, what was the reason for the implementation of postoperative RRT in the individual patients. Finally, in our study we also do not present follow-up data. Future directions of this research and unanswered questions include timing of RRT implementation and coincidence of postoperative RRT with major postoperative complications preceding this event (for example, resternotomy due to bleeding, conversion from OPCAB to CABG, severe hemodynamic instability, cardiogenic shock, postoperative cardiac arrest, etc.). Information provided by this study can make the clinicians aware of the fact, that the need for postoperative RRT is a major complication, associated with a very high mortality (>40 vs 2.2%) and therefore reasons for development should be avoided at all costs.

## Conclusion

Postoperative RRT appears more frequently among patients with preoperative co-morbidities. Preoperative renal failure and cardiogenic shock are among the most prominent risk factors for the development of postoperative RRT. This complication is associated with the increased in-hospital mortality and the presence of serious postoperative complications.

## Ethical approval

The Silesian Medical University Ethics Committee (PCN/0022/KB1/113/20 from 03 July 2020); therefore, approved this study as exempt from review and consent of patients has been waived.

## Source of funding

None.

## Author contribution

A.K.: conceptualization, methodology, resources, writing – original draft, visualization, and writing – review and editing; T.H.: resources, writing – original draft, writing – review and editing. on behalf of KROK Investigators. P.S., B.P., M.J., and T.H.: resources and writing – original draft; P.N.: conceptualization, methodology, writing – original draft, and review and editing; P.K.: conceptualization, methodology, formal analysis, Writing, original draft, review and editing, supervision, and project administration.

## Conflicts of interest disclosure

The authors have no conflicts of interest to declare.

## Research registration unique identifying number (UIN)

1. Name of the registry: Researchregistry.
2. Unique identifying number or registration ID: researchregistry10052.
3. Hyperlink to your specific registration (must be publicly accessible and will be checked): https://www.researchregistry.com/browse-the-registry#home/.

## Guarantor

Authors accept full responsibility for the work and the conduct of the study.

## Data availability statement

The personal data of patients were protected in accordance with the Polish law. The sophisticated personal data protection system provided by the National Center for Healthcare Information Systems was used during data processing. The KROK Registry is available at (www.krok.csioz.gov.pl) and does not contain any patient identifiers The Silesian Medical University Ethics Committee (PCN/0022/KB1/113/20 from 03 July 2020); therefore, approved this study as exempt from review and consent of patients has been waived.

## Provenance and peer review

Not commissioned, externally peer reviewed.

## References

[1] Swaminathan M Shaw AD Phillips-Bute BG . Trends in acute renal failure associated with coronary artery bypass graft surgery in the United States. Crit Care Med 2007;35:2286–2291. 17944016 10.1097/01.ccm.0000282079.05994.57

[2] Loef BG Epema AH Smilde TD . Immediate postoperative renal function deterioration in cardiac surgical patients predicts in-hospital mortality and long-term survival. J Am Soc Nephrol 2005;16:195–200. 15563558 10.1681/ASN.2003100875

[3] Chertow GM Lazarus JM Christiansen CL . Preoperative renal risk stratification. Circulation 1997;95:878–884. 9054745 10.1161/01.cir.95.4.878

[4] Huckaby LV Seese LM Hess N . Fate of the kidneys in patients with post-operative renal failure after cardiac surgery. J Surg Res 2022;272:166–174. 34979472 10.1016/j.jss.2021.08.025

[5] Nicoara A Patel UD Phillips-Bute BG . Mortality trends associated with acute renal failure requiring dialysis after CABG surgery in the United States. Blood Purif 2009;28:359–363. 19729906 10.1159/000235856

[6] Lenihan CR Montez-Rath ME Mora Mangano CT . Trends in acute kidney injury, associated use of dialysis, and mortality after cardiac surgery, 1999 to 2008. Ann Thorac Surg 2013;95:20–28. 23272825 10.1016/j.athoracsur.2012.05.131 PMC4115367

[7] Bonventre JV Zuk A . Ischemic acute renal failure: an inflammatory disease? Kidney Int 2004;66:480–485. 15253693 10.1111/j.1523-1755.2004.761_2.x

[8] Karkouti K Wijeysundera DN Yau TM . Acute kidney injury after cardiac surgery: focus on modifiable risk factors. Circulation 2009;119:495–502. 19153273 10.1161/CIRCULATIONAHA.108.786913

[9] Stafford-Smith M Shaw A Swaminathan M . Cardiac surgery, and acute kidney injury: emerging concepts. Curr Opin Crit Care 2009;15:498–502. 19812485 10.1097/MCC.0b013e328332f753

[10] Thakar CV Arrigain S Worley S . A clinical score to predict acute renal failure after cardiac surgery. J Am Soc Nephrol 2005;16:162–168. 15563569 10.1681/ASN.2004040331

[11] Li SY Chen JY Yang WC . Acute kidney injury network classification predicts in-hospital and long-term mortality in patients undergoing elective coronary artery bypass grafting surgery. Eur J Cardiothorac Surg 2011;39:323–328. 20739188 10.1016/j.ejcts.2010.07.010

[12] Gumbert SD Kork F Jackson ML . Perioperative acute kidney injury. Anesthesiology 2020;132:180–204. 31687986 10.1097/ALN.0000000000002968 PMC10924686

[13] Kwon JT Jung TE Lee DH . Predictive risk factors of acute kidney injury after on-pump coronary artery bypass grafting. Ann Transl Med 2019;7:44. 30906748 10.21037/atm.2018.12.61 PMC6389572

[14] Kato TS Machida Y Kuwaki K . Factors associated with postoperative requirement of renal replacement therapy following off-pump coronary bypass surgery. Heart Vessels 2017;32:134–142. 27272895 10.1007/s00380-016-0855-5

[15] Knapik P Knapik M Zembala MO . In-hospital and mid-term outcomes in patients reoperated on due to bleeding following coronary artery surgery (from the KROK Registry). Interact Cardiovasc Thorac Surg 2019;29:237–243. 30968119 10.1093/icvts/ivz089

[16] Mathew G Agha R for the STROCSS Group . STROCSS 2021: strengthening the reporting of cohort, cross-sectional and case-control studies in surgery. Int J Surg 2021;96:106165. 34774726 10.1016/j.ijsu.2021.106165

[17] Kidney Disease Improving Global Outcomes . KDIGO clinical practice guidelines on acute kidney injury. Kidney Int Suppl 2012;2:8–12.

[18] Knapik P Hirnle G Kowalczuk-Wieteska A . Off-pump versus on-pump coronary artery surgery in octogenarians (from the KROK Registry). PLoS One 2020;15:e0238880. 32913359 10.1371/journal.pone.0238880 PMC7482977

[19] Mehta RH Honeycutt E Patel UD . Impact of recovery of renal function on long-term mortality after coronary artery bypass grafting. Am J Cardiol 2010;106:1728–1734. 21126617 10.1016/j.amjcard.2010.07.045

[20] Zakeri R Freemantle N Barnett V . Relation between mild renal dysfunction and outcomes after coronary artery bypass grafting. Circulation 2005;112(9 suppl):I270–I275. 16159830 10.1161/CIRCULATIONAHA.104.522623

[21] Corredor C Thomson R Al-Subaie N . Long-term consequences of acute kidney injury after cardiac surgery: a systematic review and meta-analysis. J Cardiothorac Vasc Anesth 2016;30:69–75. 26482483 10.1053/j.jvca.2015.07.013

[22] Mathis MR Naik BI Freundlich RE . Preoperative risk and the association between hypotension and postoperative acute kidney injury. Anesthesiology 2020;132:461–475. 31794513 10.1097/ALN.0000000000003063 PMC7015776

[23] Cooper WA O’Brien SM Thourani VH . Impact of renal dysfunction on outcomes of coronary artery bypass surgery: results from the Society of Thoracic Surgeons National Adult Cardiac Database. Circulation 2006;113:1063–1070. 16490821 10.1161/CIRCULATIONAHA.105.580084

[24] Wong B St Onge J Korkola S . Validating a scoring tool to predict acute kidney injury (AKI) following cardiac surgery. Can J Kidney Heal Dis 2015;2:1–9. 10.1186/s40697-015-0037-x PMC4349478 25780626

[25] Hu J Chen R Liu S . Global incidence and outcomes of adult patients with acute kidney injury after cardiac surgery: a systematic review and meta-analysis. J Cardiothorac Vasc Anesth 2016;30:82–89. 26482484 10.1053/j.jvca.2015.06.017

[26] Axtell AL Fiedler AG Melnitchouk S . Correlation of cardiopulmonary bypass duration with acute renal failure after cardiac surgery. J Thorac Cardiovasc Surg 2020;159:170–178. 30826102 10.1016/j.jtcvs.2019.01.072

[27] Seabra VF Alobaidi S Balk EM . Off-pump coronary artery bypass surgery and acute kidney injury: a meta-analysis of randomized controlled trials. Clin J Am Soc Nephrol 2010;5:1734–1744. 20671222 10.2215/CJN.02800310 PMC2974371

[28] Reents W Hilker M Börgermann J . Acute kidney injury after on-pump or off-pump coronary artery bypass grafting in elderly patients. Ann Thorac Surg 2014;98:9–14. 24881861 10.1016/j.athoracsur.2014.01.088

[29] García Fuster R Paredes F García Peláez A . Impact of increasing degrees of renal impairment on outcomes of coronary artery bypass grafting: the off-pump advantage. Eur J Cardiothorac Surg 2013;44:732–742. 23425679 10.1093/ejcts/ezt053

[30] Wijeysundera DN Karkouti K Dupuis JY . Derivation and validation of a simplified predictive index for renal replacement therapy after cardiac surgery. JAMA 2007;297:1801–1809. 17456822 10.1001/jama.297.16.1801

[31] O’Neal JB Shaw AD Billings FT IV . Acute kidney injury following cardiac surgery: current understanding and future directions. Crit Care 2016;20:187. 27373799 10.1186/s13054-016-1352-z PMC4931708

[32] Birnie K Verheyden V Pagano D . Predictive models for kidney disease: Improving global outcomes (KDIGO) defined acute kidney injury in UK cardiacsurgery. Crit Care 2014;18:606. 25673427 10.1186/s13054-014-0606-x PMC4258283

